# “I’m quite proud of how we’ve handled it”: health professionals’ experiences of returning additional findings from the 100,000 genomes project

**DOI:** 10.1038/s41431-024-01716-6

**Published:** 2024-11-05

**Authors:** Bethany Stafford-Smith, Jana Gurasashvili, Michelle Peter, Morgan Daniel, Meena Balasubramanian, Lucy Bownass, Paul Brennan, Ruth Cleaver, Virginia Clowes, Philandra Costello, Bianca DeSouza, Louise Dubois, Rachel Harrison, Lara Hawkes, Elizabeth A. Jones, Alison Kraus, Meriel McEntagart, Suresh Somarathi, Amy Taylor, Vishakha Tripathi, Lyn S. Chitty, Melissa Hill

**Affiliations:** 1 NHS North Thames Genomic Laboratory Hub, Great Ormond Street Hospital for Children NHS Foundation Trust, London, UK; 2 Genetics and Genomic Medicine, UCL Great Ormond Street Institute of Child Health, London, UK; 3 Sheffield Clinical Genetics Service, Sheffield Children’s NHS Foundation Trust, Sheffield, UK; 4 Division of Clinical Medicine, University of Sheffield, Sheffield, UK; 5 Clinical Genetics, St Michael’s Hospital Bristol, University Hospitals Bristol NHS Foundation Trust, Bristol, UK; 6 Northern Genetics Service, Newcastle Hospitals NHS Foundation Trust, Newcastle, UK; 7 Peninsula Clinical Genetics Service, Royal Devon University Healthcare NHS Foundation Trust, Exeter, UK; 8 North West Thames Regional Genetics Service, Northwick Park and St Mark’s Hospital, London, UK; 9 Wessex Clinical Genetics Service, Princess Anne Hospital, Southampton, UK; 10 Liverpool Centre for Genomic Medicine, Liverpool Women’s NHS Foundation Trust, Liverpool, UK; 11 Department of Clinical Genetics, Nottingham University Hospitals NHS Trust, Nottingham, UK; 12 Oxford Centre for Genomic Medicine, ACE building, Nuffield Orthopaedic Centre, Oxford, UK; 13 Manchester Centre for Genomic Medicine, St Mary’s Hospital, Manchester University NHS Foundation Trust, Manchester, UK; 14 Division of Evolution, Infection and Genomics, School of Biological Sciences, Faculty of Biology, Medicine and Health, University of Manchester, Manchester, UK; 15 Yorkshire Regional Genetics Service, Chapel Allerton Hospital, Leeds, UK; 16 Medical Genetics, Clinical Developmental Sciences, St. George’s University of London, London, UK; 17 Clinical Genetics Unit, Birmingham Women’s and Children’s NHS Foundation Trust, Birmingham, UK; 18 Clinical Genetics, East Anglian Medical Genetics Service, Cambridge, UK; 19 Department of Clinical Genetics, Guy’s and St Thomas’ Hospitals NHS Trust, London, UK

**Keywords:** Social sciences, Health care

## Abstract

Participants in the 100,000 Genomes Project (100kGP) could consent to receive additional finding (AF) results, individual variants relating to genes associated with susceptibility to cancer and familial hypercholesterolemia (FH). In the study reported here, qualitative interviews were used to explore the experiences of National Health Service (NHS) professionals from across England who were tasked with returning over 80,000 “no AF” results and 700 positive AF results to 100kGP participants. Interviews were conducted with 45 professionals from a range of backgrounds, including Genetic Counsellors, Clinical Geneticists, FH Clinical Nurse Specialists and Clinical Scientists. Interviews were analysed using a codebook thematic analysis approach. Returning AF results has been a significant endeavour, with challenges for pathways, administrative processes and clinical and laboratory time when the capacity of NHS services is already stretched. Professionals discussed going “above and beyond” to prioritise patient care through pathway design, additional clinics, overtime, longer appointments and provision of follow-up appointments. Professionals also described facing practical and emotional challenges when returning AFs. Benefits for patients from receiving AFs in the 100kGP were highlighted and professionals were generally positive about offering clinically actionable AFs within routine NHS clinical care. Professionals were, however, cautious around the implementation of AFs into routine care and felt more research and discussion was needed to determine which AFs to offer, approaches to consent and communication of results, costs and the potential strain on NHS capacity and resources. Further consultation is required with careful review of pathways and resources before offering AFs in clinical practice.

## Introduction

Genome sequencing (GS) is transforming modern healthcare by improving the diagnostic yield of rare disease and providing information on cause, prognosis, and therapeutic impact for some cancers. When GS is performed there is an opportunity to look for health related “additional findings” (AFs), also called “secondary findings”, that are unrelated to the patient’s primary indication for GS testing. The goal of offering AFs is to identify a possible increased risk for conditions that the individual may not be aware of that will allow patients to be proactive in reducing risks and sharing information with family members. There is, however, ongoing discussion around whether and in what circumstances AFs should be offered, and how to offer AFs in a way that balances benefits and minimises potential harms [1–5]. Considerations include the potential burden of unwanted information and the clinical value of the information, especially in the absence of a relevant family history [1–5]. Consensus from professional bodies is lacking. Current American College of Medical Genetics and Genomics (ACMG) guidelines recommend returning pathogenic and likely pathogenic variants in 73 genes [6], while guidelines from other countries are more conservative [4, 7, 8]. Moreover, while previous research shows patients, clinicians and the public support reporting medically actionable AFs [9–11] and a recent systematic review found no evidence of negative psychological impacts on patients [12], reported processes for consent and return of results and the type of AF reported vary widely [5].

**Fig. 1:**
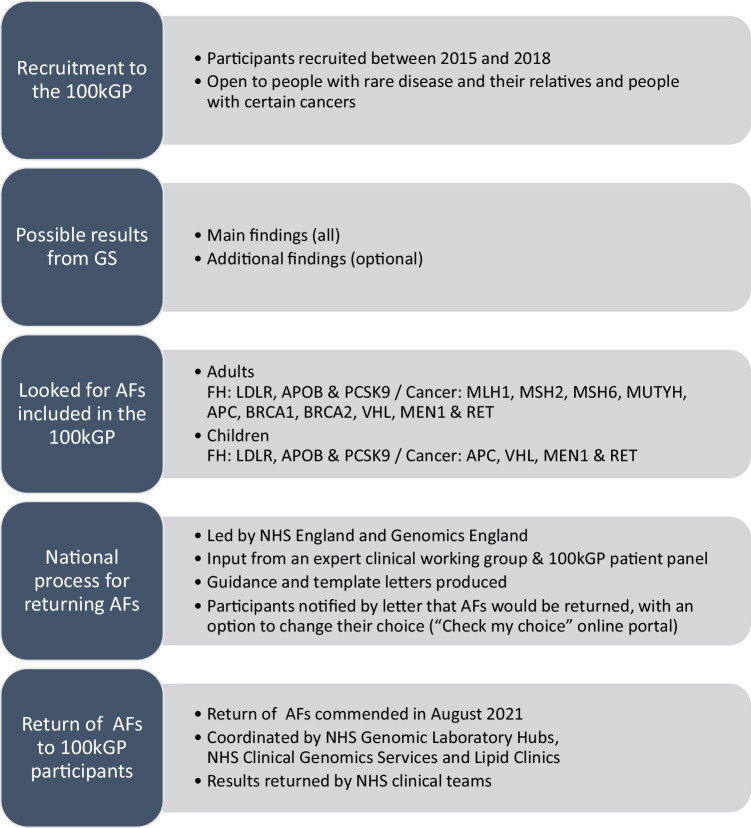
Overview of the national process for returning AFs from the 100kGP.

In England, GS has been offered in routine care since 2020 through the National Health Service (NHS) Genomic Medicine Service [13]. AFs are not routinely offered to patients who have GS as more evidence is needed to guide whether and how AFs should be offered. The NHS Genomic Medicine Service was largely informed by the 100,000 Genomes Project (100kGP) [14, 15]. Between 2015 and 2018 over 85,000 patients with cancer or rare disease, and their relatives, were recruited to the 100kGP (Fig. 1). All participants consented to receive main findings relating to their cancer or rare disease and had the option to consent to receive clinically actionable AFs for 13 genes associated with an increased risk of some cancers or familial hypercholesterolaemia (FH) [16]. AFs for children were a subset of seven of these 13 genes, with adult-onset conditions omitted. Consent was taken by professionals from a range of backgrounds, including genetic counsellors and research nurses, who had undergone 100kGP consent training [17].

Main findings were returned to 100kGP participants by the referring NHS clinical teams as results became available. After main findings were returned, a unified national process was established to return AFs to 100kGP participants. Over 90% of 100kGP participants had opted to receive AFs and more than 700 positive AFs (PAF) results and 80,000 no AF (NAF) results have been returned through NHS pathways. Recent research in one English region has shown that the identification of patients with PAF results has enabled appropriate clinical interventions [18].

Here we have used qualitative interviews with professionals to explore their experiences of returning AFs and gather their views on offering AFs in routine care. This study is part of a broader evaluation of the clinical, behavioural, psychological and economic impacts of returning AFs to 100kGP participants. Findings relating to patient experiences and to costs will be reported separately.

## Methods

### Study design

Qualitative semi-structured interviews were used to facilitate an in-depth exploration of professionals’ views and experiences.

### Setting

The setting is the return of AFs from the 100kGP in England. Starting in August 2021, AFs were released by Genomics England to local services in batches that were several weeks apart. National guidance and template letters were produced to guide local processes for returning AFs. All NAF results were sent out by letter. The suggested pathway for PAF results was to send a notification letter that explains a PAF has been found with an invitation to a clinical appointment where the condition will be disclosed. The suggested maximum time between notification and disclosure was six weeks. Standard NHS pathways for ongoing care were then followed, including recommendations for risk management and cascade testing.

### Sampling and recruitment

Professionals from NHS Trusts, Genomics England and NHS England involved in planning, overseeing and delivering the return of AF results to 100kGP participants were purposefully sampled to include participants from different geographic locations and professional backgrounds. Potential participants were identified by the research team and invited to take part via email.

### Interviews

Interview topic guides explored: (1) Views on offering AFs to 100kGP participants, (2) Local pathways, processes and challenges, (3) Experiences of returning AFs and (4) Views on offering AFs in routine practice (Supplementary Materials). Interviews were conducted via telephone or video call.

### Data analysis

Interviews were digitally recorded, transcribed verbatim and pseudonymised. Data were analysed using the principles of thematic analysis [19] with a team-based codebook approach [20]. NVivo version 13 (QSR International Ltd) facilitated coding. Inductive and deductive codes were used to develop the codebook [21]. BSS drafted an initial codebook based on study aims and topic guides (deductive codes). The draft codebook was inductively revised by BSS, JG and MH who independently coded three transcripts and added additional codes (inductive codes). The final codebook was then used to code all transcripts. Additional inductive codes were added throughout the coding process.

## Results

### Participant characteristics

Of 65 professionals invited by email to participate, six did not respond, 14 declined and 45 participated (response rate: 69%). Interviews were conducted between May and October 2022 (seven by telephone, 38 by video call), by BSS (*n* = 8), JG (*n* = 10) and MH (*n* = 27) and lasted between 26 and 70 min (median = 43 min). Participants included genetic counsellors (53%), clinical geneticists (20%), specialist FH nurses (16%) and clinical scientists (7%) working across England (Table 1). The majority of participants had direct experience of returning PAFs to patients (32/45), with others involved in administration, coordination or laboratory work.

**Table 1:** Participant characteristics.

Professional role		
Clinical geneticist	9	20%
Genetic counsellor or nurse	24	53%
Consultant endocrinologist	1	2%
FH nurse specialist	7	16%
Clinical scientist	3	7%
Service Manager	1	2%
Region in England		
North West	4	9%
North East and Yorkshire	6	13%
East	4	9%
Central and South	16	36%
North Thames	6	13%
South East	4	9%
South West	3	7%
NA	2	4%

### Interview findings

Findings are described within three overarching themes:

1. Pathways and processes for returning AFs from the 100kGP
2. Experiences of returning AFs from the 100kGP
3. Views on offering AFs in routine care

### Pathways and processes for returning AFs from the 100kGP

#### Variation in pathways and processes

**Fig. 2:**
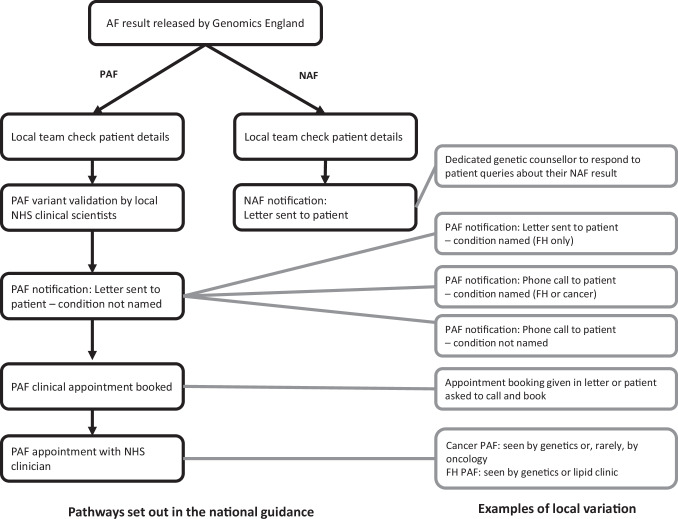
National pathway and identified local variation when returning AFs in the 100kGP. AF additional finding, PAF positive additional finding, NAF no additional finding, FH familial hypercholesterolaemia.

Care pathways and template letters were adapted from the national guidance by some services (Fig. 2). One key difference between local pathways for returning PAFs was the approach for disclosing the condition. Some teams disclosed the condition in a notification letter (FH only) or in a notification telephone call (cancer or FH), while others waited until the subsequent clinical appointment (cancer or FH). Disclosing the condition in the notification letter or telephone call was chosen to minimise anxiety while patients waited for their clinical appointment (Table 2: Q1). The types of clinicians involved in returning AFs varied between local services (Fig. 2). Sending out NAF results letters was managed by administrative teams. Some professionals reported receiving patient queries about their NAF result letter and some services had assigned a genetic counsellor to provide support and answer questions from participants with NAF results by telephone (Table 2: Q2).

**Table 2:** Pathways and processes for returning AFs from the 100kGP.

Quote number	Illustrative quote
*Variation in pathways and processes*	
Q1	“We made the decision to use bespoke letters telling the patients what their additional finding was, because I think that was probably less stressful for the patient.” *HP31 - FH Clinical Nurse Specialist*
Q2	“We’re still getting patients who are calling saying, “What’s this all about?” or, “Why have I only just heard about this?” So it’s still caused upset… we decided to put a genetic counsellor in place to field those sorts of calls.” *HP03 - Genetic Counsellor*
*Going above and beyond to prioritise patient care*	
Q3	“At every stage that we were planning it we were thinking OK what would be best for that patient.” *HP01 – Genetic Counsellor*
Q4	“So I’m quite proud of how we’ve handled it, there’s been a lot of flexibility… lots of going above and beyond, you know, speaking to patients in the evenings when they finished work because that’s the time that they can talk, you know, arranging ad hoc appointments, arranging extra follow up appointments as extras on the end of clinics, you know, I think there’s been a lot of energy and effort that has gone into these.” *HP21 – Genetic counsellor*
*Practical challenges*	
Q5	“You can’t just find an extra geneticist or you can’t just find an extra lab scientist who can interpret things. But I think that’s a common thing across the country isn’t it? Everybody’s struggling to get the right staff. And I think just on top of Covid it’s just made it all much worse.” *HP27 - Clinical Geneticist*
Q6	“The actual delivery of this did take away from our routine clinical time at a difficult time… sorting out the funding and then sorting out the people to do this work was difficult at the time it was – the way it clashed with Covid at the time that the team was very, very stressed and our waiting lists had never been higher” *HP13 – Genetic Counsellor*
Q7	“In the 100 K the primary findings went back to [the mainstream clinicians] and weren’t always conveyed to patients or a referral to genetics triggered.” *HP07 – Genetic Counsellor*
Q8	“So that was just telling them something they already knew, but of course it still had created anxiety, but of course relief when it was something they were already aware of.” *HP16 - Genetic Counsellor*
Q9	“When I had that appointment her first thing was “Well has my mother got this BRCA1 variant?” And her mother hadn’t received anything. So I did have to say “Unfortunately not all the letters are going out together so your mother’s just going to have to wait and she’ll get a letter telling her either nothing’s been found or something has”… So that’s been quite hard with letters going out at different times.” *HP33 – Genetic Counsellor*

#### Going above and beyond to prioritise patient care

Many professionals described how their teams went “above and beyond” to prioritise patient care, highlighting that “flexibility” and “working around the patient” were key to constructing local pathways (Table 2: Q3). Approaches included: pathway planning to minimise delays between notification letter/telephone call and clinic appointment, additional clinics (including evenings or weekends), telephone helplines, development of guidance for counselling, longer appointments and overtime (paid and unpaid). Many highlighted the value of close team working to effectively return AFs. Regular team meetings were valued as a space to “troubleshoot any problems, if we’ve had any issues disclosing the result or if it’s been a particularly psychosocially challenging consultation then that’s the space really where we can share thoughts, concerns and ideas” (HP04 – Genetic Counsellor). Successfully establishing pathways and addressing challenges that would allow teams to provide the best possible care for patients gave professionals a sense of pride (Table 2: Q4).

#### Practical challenges

The sheer number of results to be returned meant that “one of the biggest challenges was just the huge amount of admin involved” (HP45 – Clinical Scientist). Clinical scientists noted challenges arising from the time required for variant interpretation and the lack of standardised software across different laboratories. While some professionals felt their team had sufficient capacity, many commented that clinical and laboratory services were already strained due to COVID-19 (Table 2: Q5). It was also noted that returning AFs within the set timeframes was “something additional” that impacted existing workloads and the delivery of routine care (Table 2: Q6). Some participants added that “these patients get pushed in front of others” (HP16 – Genetic Counsellor), increasing routine waiting times. Conversely, one FH nurse noted that COVID-19 had reduced referrals for FH testing, which meant that patients identified through the 100kGP “filled a gap” and were not a burden on capacity. The time between recruitment and returning AFs meant that local knowledge and experience of the 100kGP was reduced because the dedicated “infrastructure and staff had moved away” (HP37 – Clinical Scientist).

Other issues added to demands on capacity and resources. For example, initiating the return of AFs highlighted that not all 100kGP participants had received their main findings due to gaps in coordination and communication (Table 2: Q7). Some patients already knew of their increased risk for the condition identified as a PAF, because the PAF was the same as their main finding or because the condition had already been identified through standard clinical pathways (Table 2: Q8). Finally, returning results in batches that did not include all family members could generate anxiety for some patients and required additional administrative and clinical time (Table 2: Q9).

### Experiences of returning AFs from the 100kGP

#### Returning AFs to 100kGP participants was viewed positively

Benefits of returning AFs to 100kGP participants primarily centred on the included conditions being recognised as actionable with clear clinical benefits for patients (Table 3: Q1). For cancer, the value of facilitating access to screening and early diagnosis was frequently noted (Table 3: Q2). For FH, the importance of identifying patients who can have a “simple treatment” to reduce their risk of cardiovascular disease (Table 3: Q3) made FH a “good example of minimal psychological harm – massive benefit” (HP06 – FH Clinical Nurse Specialist). Another frequently noted benefit was cascade testing to identify at risk family members. Professionals who had returned results to 100kGP participants described psychosocial benefits for patients, as some patients felt “empowered” to make choices about their healthcare (Table 3: Q4) and some patients expressed relief that their family history of cancer or FH now made sense.

**Table 3:** Experiences of returning AFs from the 100kGP.

Quote number	Illustrative quote
*Returning AFs to 100kGP participants was viewed positively*	
Q1	“I think for 99% of people it’s a very useful thing and I think people are generally interested in their risks, especially the people who have been recruited into such a process and they are often motivated to take action.” *HP17 – Genetic Counsellor*
Q2	*“T*he ones that I’ve been involved in so far are convey a cancer predisposition syndrome, we can put in place screening that the families would not have been able to access or even been aware of before this, so I see that as the biggest advantage to these.” *HP04 – Genetic Counsellor*
Q3	“The patients that we’ve seen we’re able to make sure that they have their cholesterol levels done, that they get referred to a lipid clinic, that they get started on treatment. So long-term reducing their risk of cardiovascular disease… I think that’s the real benefit long term for both children and adults.” *HP22 - FH Clinical Nurse Specialist*
Q4	“Having had a number of these appointments now and given these results to people, I think they have all given patients information which they have felt was very useful and actionable and I think it’s information that has the potential to really empower patients and to provide them with some knowledge of something that they didn’t have before” *HP36 – Genetic Counsellor*
*Returning AFs felt “out of the blue” for patients and professionals*	
Q5	“I think part of the problem is the delay, so people have forgotten almost that they’ve done it all, it’s gone to the back of their mind.” *HP16 – Genetic Counsellor*
Q6	“So, the results that I’ve delivered, the first gentleman I mentioned was highly anxious, thought I was going to tell him that he was going to get early onset dementia so he completely misunderstood what additional findings were being looked for.” *HP08 – Genetic Counsellor*
Q7	“Just doing it bit by bit felt more compassionate and careful and safe as well. I think there’s only so much that a person can take in during an appointment and particularly when it’s one like this where they’ve not had any heads up.” *HP04 – Genetic Counsellor*
Q8	“We knew nothing about the patients, you know, at least when we get a referral form from a GP you get a little bit of history, you get their cholesterol results, you get are they on statins, have they got any family history, have they got any personal clinical history of cardiac disease. But for these patients, we know nothing about them. We know that they’ve been recruited for another reason so they’ve had a traumatic journey of some description, whether it be cancer, rare disease in a child or in themselves” *HP31 – FH Clinical Nurse Specialist*
Q9	“And then I had a quiet chap who was he was from Poland and his English was pretty good, but he was a very gentle man, he absorbed the information and it was hard to glean from him what he felt emotionally about it… we’d not had an opportunity to build up a rapport with him and, I must admit, I felt quite bad giving him that result with him not knowing our service personally, giving it out of the blue after six/seven years” *HP13 – Genetic Counsellor*
*Complex and challenging clinical scenarios*	
Q10	*“*I think one of the biggest difficulties is interpreting what this means for these families where there’s no family history of an associated problem and making a meaningful interpretation of what their result, how the result is going to impact them in terms of risk and what the most appropriate surveillance or surgical options we best advise on.” *HP04 – Genetic Counsellor*
Q11	“So it’s been tricky to counsel people a bit because you have to say well yes, we quote these really high risks of breast cancer say that actually that might be different in your family because we can see that you’ve not got a strong family history of this at all. So it’s whether we’re sort of raising anxiety even though, because we’d never have offered this test anyway.” *HP33 – Genetic Counsellor*
Q12	“We’ve had some people who have said they don’t want to know. So then you’ve got, sort of, a responsibility to that patient and to their family but it is hard for you to discharge that responsibility. When the person is dead then you don’t know their next of kin, again you can’t discharge your responsibility and I think that causes a clinical concern because people don’t like sitting on information that they can’t pass on.” *HP05 – Clinical Geneticist*

#### Returning AFs felt “out of the blue” for patients and professionals

Professionals reported that the three to six years between consent and results meant that many patients had either forgotten or had limited recall of the consent discussion (Table 3: Q5). There were also misunderstandings about what conditions had been looked for (Table 3: Q6). Consequently, AF results came “out of the blue” or were “quite a shock” and often meant patients “were quite anxious… not just for themselves but also for the wider family” (HP08 – Genetic Counsellor). The disclosure appointment was sometimes described as “overwhelming” for patients, particularly for cancer AFs. Follow-up appointments allowed patients to “regroup” and “digest it in a calmer way” (Table 3: Q7). Professionals described supporting patients to enable a good understanding of their risk and management options, which allowed them to feel “back in control” (HP09 – Clinical Geneticist).

As many patients were previously unknown to the clinical service, professionals had not met the patient before or did not have access to their clinical notes (Table 3: Q8). Several professionals commented that their usual approach to supporting patients through genetic testing that involved pre- then post-test counselling was reversed which “felt all a little bit, I don’t know, out of the blue for me and for them” (HP04 – Genetic Counsellor). Discussions that usually occur during pre-test counselling, such as describing test implications and taking a family history, were incorporated into the results appointment. Some noted that when patients have timely pre-test counselling it is clear why a genetic test is indicated and “expectations are managed much better” (HP34 – Genetic Counsellor). In addition, without the opportunity to build rapport through pre-test counselling, it could be difficult to gauge the patient’s emotional response to the AF results (Table 3: Q9).

#### Complex and challenging clinical scenarios

Several clinical scenarios arose that professionals found practically and emotionally challenging. Many highlighted the difficulty of interpreting the risk for a participant with a PAF when there was no family history (Table 3: Q10). Professionals described balancing “finding something and then being able to offer patients screening which would be a good thing versus the worry that might cause when there’s no family history” (HP44 – Genetic Counsellor). In addition, explaining the uncertainty around the level of risk for the patient and their family members was described as “challenging” or “tricky” (Table 3: Q11). Several professionals also noted the “clinical quandary” of dealing with AF results released for 100kGP participants who were deceased with no recorded next of kin or relatives said they did not want the results (Table 3: Q12). Knowing they had information of value that they were unable to share left professionals unsure of next steps and concerned that they could not discharge their responsibilities. Emotionally challenging scenarios included sharing cancer AF results with participants who had developed cancer after consenting for the 100kGP or with participants who were currently pregnant. These situations were distressing for patients and their families, and professionals described feeling frustrated and upset themselves.

### Views on offering AFs in routine care

#### Cautious optimism for offering AFs more widely

Professionals generally felt that including AFs routinely when GS was offered would be a “positive step” and described a range of potential benefits but also highlighted multiple practical and resource challenges to overcome (Table 4: Q1). Accordingly, many professionals felt that further evidence was needed. For example, establishing a list of clinically actionable AFs that aligns with the availability of NHS resources to action them, and more evidence to accurately interpret risks when there is no family history of the condition. Many also noted that further consultation with stakeholders was needed to inform the individual specific decisions about whether AFs should be included routinely as well as how, when and to whom they should be offered. One professional with experience of bringing new genomic tests into clinical practice noted the importance of gauging acceptability amongst a wide range of key stakeholders, including clinicians, clinical scientists, patients and the public because “people that are part of a research project have a very different view to people who might just be coming as part of a clinical service” (HP41 – Service manager).

**Table 4:** Views on offering AFs more widely.

Quote number	Illustrative quote
*Cautious optimism for offering AFs more widely*	
Q1	*“*I can see a number of benefits to offering it… we’re going to learn a lot about revised penetrance figures for these conditions… and I think with the right support patients, I do think patients feel empowered to be able to access that screening and to make choices about their healthcare going forward. However, I do just think it needs to be acknowledged the potential for harm within that and I think that needs to be, those decisions around offering that routinely need to be made very, very carefully and ensuring that the right pre-test and post-test support is there and that that’s something that clinic genetic services have capacity to manage before that’s implemented.” *HP21 – Genetic Counsellor*
Q2	*“*I think from a workload perspective… how are we going to see all these patients and how are we going to find the time… I think if we’re sending out these results then we really do need people on hand to take calls from patients and to be very responsive to dealing with their anxiety if they are calling in and are really struggling.” *HP36 – Genetic Counsellor*
Q3	“We have referrals for patients with family histories of breast cancer and we decline them because they don’t meet the eligibility criteria for having the test… If we’re going to offer it to other patients who don’t have a family history of breast cancer, we have to first offer it to every patient with a family history of breast cancer…and same for bowel cancer and the same for any of the other conditions.” *HP02 – Clinical Geneticist*
Q4	“I think the whole issue around children will be difficult because obviously in FH we do test children when we treat children, but if you find out you’ve got a BRCA mutation and you’ve got a ten year old, well we’re not going to test that ten year old and I think that could be quite distressing for a lot of families actually, to know that it’s going to be a while before their child can be tested, things like that. So, I don’t think it’s a bad thing by any stretch of the imagination, but I just think there needs to be a lot of thought around it before it starts, definitely.” *HP22 - FH Clinical Nurse Specialist*
*Careful curation of the specific AFs to offer is needed*	
Q5	“I just think high penetrant genes generally where there is a clear evidence base and management plan that you can put in place that has been shown to reduce the risk associated with a particular condition, so it has to be a condition where you can take steps to reduce your risk of developing adverse effects from said condition.” *HP15 – Clinical Geneticist*
Q6	“It’s not whether we can, it’s whether we should. And that, for me, depends heavily on what does it mean for a family, what can we do for that family, because if it’s just information, that’s not good enough for me, it’s got to be we’ve found this, now we can do this and now we can offer this, and this will help because…” *HP19 – Genetic Counsellor*
*Approaches to taking consent for AFs need careful consideration*	
Q7	“It is important that they have the competencies and skills, I don’t think it really matters what type of healthcare professional they are, whether they’re a GP, a nurse, a counsellor, a paediatrician, I think it’s more about the competencies that they have… I think maybe it should be the specialities that do divulge that so that they know the sort of questions, the answers, the worries or concerns, next steps, processes that they need to anticipate and explain to the patient. *HP06 - FH Clinical Nurse Specialist*
Q8	“If this was broadened out then it would have to be the mainstreamers, whether that would be the breast surgeons or oncologists or whoever, they would have to be able to deal with it and maybe we would need some MDT work to discuss how a variant is going to be interpreted and what a plan is going to be.” *HP05 – Clinical Geneticist*

Benefits for offering AFs routinely included providing patients with clinically actionable findings, screening, information for management, cascade testing, earlier detection and treatment and insights into gene penetrance. The most common concern was whether the NHS had the capacity and resources to manage the additional workload for clinicians, clinical scientists and administrative staff. Many professionals felt the NHS is already “stretched,” “swamped,” and “not set up to deliver on this scale” and questioned if it would be feasible to add AFs: “who’s going to fund it and who’s going to see them” (HP22 – FH Clinical Nurse Specialist). A linked concern was how to provide the necessary psychological support for patients (Table 4: Q2). The issue of costs relative to benefits was raised and concerns around equity of access to genetic testing were also discussed. Some professionals noted the inequity of offering tests to patients without a family history ahead of patients with concerns around their family history who fall short of current eligibility criteria which requires a strong family history of cancer (Table 4: Q3). Several professionals also noted the “ethical tensions” around offering AFs to paediatric patients around what to report, holding the information, the timing of the disclosure for actionable variants in adult-onset genes and distress for parents unable to access some tests for their child until adulthood (Table 4: Q4).

#### Careful curation of the specific AFs to offer is needed

All professionals felt that if AFs were to be offered routinely, then the list of genes and variants would need to be carefully curated to include only actionable findings with a clear pathway for surveillance and risk management (Table 4: Q5). For example, some clinicians worried about including genes where screening is not universally available across the UK (e.g., *TP53*). Several professionals also strongly felt that “ambiguous results”, such as genetic alterations in low penetrance genes and variants of uncertain significance, should not be offered (Table 4: Q6). Many professionals thought that the 13 genes offered in the 100kGP would be a good starting point. Most felt the current ACMG list was too extensive, holding concerns that not all ACMG genes were truly actionable within the NHS and that the “infrastructure to deal with it” (HP28 – Clinical Scientist) is lacking.

#### Approaches to taking consent for AFs need careful consideration

Professionals discussed possible consent processes for offering AFs in clinical practice. Many professionals felt that consent should be specific to AFs, rather than how it was offered in the 100kGP, with a “tick-box” added onto an existing consent form. Some participants acknowledged that a more in-depth consent process would be ideal, but it may not be feasible due to limited resources. Offering AFs alongside GS in acute care settings, such as rapid sequencing for acutely unwell children, was flagged as requiring more thought as parents may not be able to fully attend to the implications. A two-step consent conversation was viewed positively to separate decisions about clinically indicated testing and AFs, to allow patients to “deal with what they need to deal with and when they’re ready they can think about anything further” (HP16 – Genetic Counsellor). Approaches where patients “opt out” of receiving AFs were not thought to be appropriate.

While many felt that appropriately trained professionals from a range of backgrounds could offer and consent for AFs, returning results needs to involve specialists in the condition or clinical genetics (genetic counsellors and clinical geneticists) experienced in explaining next steps for management and referrals (Table 4: Q7). While some professionals felt strongly that returning AFs should remain with genetics teams who are “much more used to dealing with families,” others felt that the shift to embed mainstreaming and the value of multidisciplinary team (MDT) working meant that integrating the return of AFs across relevant health disciplines would “make the most sense”. MDT working requires coordinated care pathways to allow mainstream teams to access support from genetics when needed (Table 4: Q8).

## Discussion

**Fig. 3:**
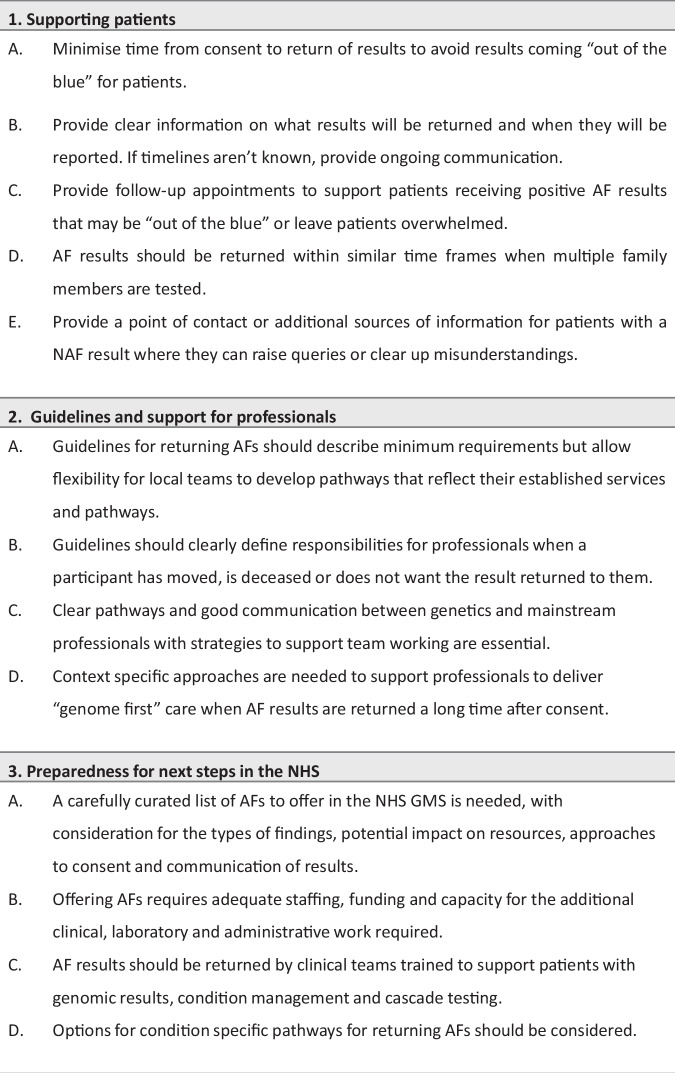
Summary of lessons from professionals involved in returning AFs from the 100kGP. Key: AF additional finding, NAF no additional finding.

The return of AFs through NHS clinical care pathways from a research project with the expansive scale of the 100kGP provides a unique opportunity for insight for offering GS in both research and clinical settings. Interview findings have been considered against the wider literature and the identified lessons are summarised in Fig. 3. Patient-centred care has been prioritised throughout the process of returning AFs. Practical challenges primarily related to the large number of AFs to be returned when clinical and laboratory services are already stretched. Some teams struggled with the additional workload and reported impacts on routine patient care. These findings align with previous studies highlighting the potential for tension between research and clinical practice [22, 23], including the challenges of disclosing research results when there are limitations in infrastructure and staffing [23]. Timelines of several years between consent and return of results brought multiple challenges including patients’ poor recall of consent, results coming “out of the blue”, unclear responsibilities when a participant was deceased and emotionally charged interactions when patients were pregnant or had already developed the condition identified as a PAF. In another study, qualitative interviews with 100kGP participants with a PAF found that some had incomplete recall or misunderstandings about consent and most were surprised or shocked to receive their PAF result [24]. In addition, earlier research looking at consent experiences in the 100kGP found that some participants had misunderstood or could not remember whether they had opted to receive AFs [25, 26]. Research exploring the lessons from returning AFs in eMERGE also highlight similar challenges for consent and return of results and minimising the time between testing and reporting was suggested [27] (Fig. 3: Lesson 1A). If timelines for returning results are not clear at the outset, which was the case for the 100kGP, pathways for ongoing communication with participants are essential (Fig. 1: Lesson 1B). Guidance for professionals is needed that provides clarity on their responsibilities for deceased patients or those who do not want the result (Fig. 3: Lesson 2B). The potential for emotional burden for professionals also needs to be addressed, with time to process the experience and space for reflective practice [28].

Several participants noted that offering AFs in the 100kGP differed from the traditional genetic counselling model of pre- and post-test counselling appointments conducted by the same professional and closely spaced in time. For 100kGP participants who had consented to receive AFs several years ago, AF results were often unexpected, and results were rarely returned by the clinician who had recruited them. This experience aligns with a “genome first” approach to results disclosure where research participants are referred to clinical services after GS testing has been performed [29, 30]. Professionals in our study described how they supported patients with this model of care by adapting the traditional content of disclosure appointments to include elements of pre-test counselling, such as gathering family history information. They also emphasised the importance of follow-up appointments which allowed patients time to process information and return with further questions (Fig. 3: Lesson 1C). When AF results are returned after a long time or by clinicians not known to a patient, context specific guidance is needed for genome first counselling that considers adapting risk assessments, counselling content and information materials [30] (Fig. 3: Lesson 2D).

Returning AFs from the 100kGP will inform NHS practice. Professionals noted potential benefits of offering a curated list of medically actionable AFs (Fig. 3: Lesson 3A), but also highlighted the need for further research evidence and consultation with key stakeholders (e.g. patients, clinicians, clinical scientists and policy makers) around what AFs to offer and to whom, processes for consent and communicating results, provision of sufficient staff to support patients and potential strain on resources in an already stretched NHS. Our findings echo previous research conducted with professionals involved in 100kGP recruitment who supported disclosing a limited number of highly predictive and medically actionable AFs and raised concerns around how capacity and funding would be provided [2, 3] (Fig. 3: Lesson 3B). Our findings also align with ongoing discussions about the need to determine best practice for consent with consideration for options such as “broad consent” [27] or “dynamic consent” that allow for changes over time [10] or “two step” approaches where the offer of AFs is made after diagnostic testing [31]. Appropriate pathways for communicating results must also be addressed, including: whether to return AFs separately to main findings and consideration for how to support people with NAF results. In this study, some teams had a genetic counsellor available to discuss queries about NAF results by phone, other strategies to consider include developing resources such as websites or videos to answer frequently asked questions [32] (Fig. 3: Lesson 1E). Considerations for offering AFs must be set against the challenges the NHS faces in offering GS generally, with recent research highlighting the lack of a trained and available workforce of clinicians and scientists, and the need for improved digital infrastructure [33].

Mainstreaming GS requires new pathway development for consent and results and consideration for the roles of mainstream and genetics professionals. Establishing clear pathways and good communication between mainstream and genetics teams will be essential (Fig. 3: Lesson 2C). Notably, the type of staff involved in returning PAF results differed between services and professionals emphasised the value of MDT working. The role of lipid clinics in returning FH AFs highlights the value of condition specific approaches to returning AFs, with established pathways deployed to support patients (Fig. 3: Lesson 3D). Our findings also demonstrate the need for flexibility in future guidance to allow local adaptation to suit existing infrastructure, care pathways and skill sets, with minimum standards defined (Fig. 3: Lesson 2A). Reaching agreement on what constitutes “best practice” in genomic healthcare and how this is applied in clinical practice by local teams can be challenging, especially as new demands must be applied within existing contexts [34–36]. Previous research addressing the role of ambivalence has highlighted the importance of open discussions and additional voices, including those of patients and the public, to help interpret and inform local practices [34]. As such, broad consultation will be a crucial next step in decision making about offering AFs in clinical practice.

### Strengths and limitations

A key strength was that participants were recruited across England, had a range of professional backgrounds and differing roles in returning AFs. As interviews took place during the process of returning AFs, we have captured experiences in real time, however this may have limited time for reflection. This was a relatively small qualitative study within the specific setting of the 100kGP and NHS clinical practice which may not be generalisable to other settings. The small sample size prevented sub-group comparisons. Professionals chose to take part, potentially introducing self-selection bias as they may hold differing views and experiences to the professionals who choose not to take part. Additionally, the researchers naturally and unintentionally may introduce their own inherent bias from their experience in genomics, personal beliefs and cultural backgrounds, however the researchers engaged in reflexivity to help reduce such bias [37].

## Conclusions

As considerations are made about incorporating AFs into routine care when GS is offered, our findings provide valuable information for the design and delivery of care pathways offering AFs in research and clinical settings. Prior to routine implementation in the NHS, further consideration is required around which AFs to offer, the consent process, approaches to communicating results, and managing the increased demand on NHS laboratory and clinical services. Future guidance will benefit from the flexibility to allow local adaptations to existing pathways, infrastructure and roles. This study also highlights the need for tailored support for patients receiving unexpected results and the importance of timely results.

## Supplementary information

Additional Findings Study – topic guide for health professional interviews

## Data Availability

The data that support the findings of this study are available from the corresponding author (MH) upon reasonable request and where participant consent has been given.
