# Supplementary material for: “I’m quite proud of how we’ve handled it”: health professionals’ experiences of returning additional findings from the 100,000 genomes project
Source: Eur J Hum Genet. 2024 Nov 5;33(8):1025–34. doi: 10.1038/s41431-024-01716-6 (PMC12322091; doi:10.1038/s41431-024-01716-6)
Supplement: Supplementary file 1 — Additional Findings Study – topic guide for health professional interviews [file 41431_2024_1716_MOESM1_ESM.docx]

# Additional Findings Study – topic guide for health professional interviews

**Background**

1. Can you start by giving me a little background about yourself and your clinical role?
2. Prior to returning additional findings, were you involved in the 100,000 Genomes Project?
3. What other experience do you have of offering exome and genome sequencing to patients?
4. [non-genetics professionals only] Have you had an opportunity to complete any training or education modules specifically about genetics or genomics?

**Views on offering additional findings in the 100,000 Genomes Project**

1. Thinking broadly, what do you see as the main benefits of offering looked for additional findings to patients who opted to receive them in the 100,000 Genomes Project?
2. Do you have any general concerns offering patients looked for additional findings as part of the 100,000 Genomes Project?

**Local pathways, processes and challenges**

1. [Clinical scientists only] Can you talk me through an overview of the steps you follow to accept a case and get to the point of returning a result?
2. Have the local links and care-pathways between non-genetics, genetics and the lab teams been effective?
   - Did you have regular MDT meetings to support this?
   - Was there anything you thought could have improved this process?
3. Can you guide me through the overall approach taken to returning positive additional findings results in your region?
   - How are patients first told they have an additional finding? E.g. letter / phone call
   - How are they first told what the result is?
   - What appointments do they have – what is the background of the clinician?
4. What approach was taken when returning “no findings” additional findings results?
   - What information were patients with a ‘no findings’ result provided with?
   - Did patients have a number to contact with questions
5. What approach was taken to re-consenting young people to the 100,000 Genomics Project?
6. Thinking about this process overall;
   - How was it first decided this would be the approach used? (eg meetings of local genetics team/discussion with wider teams)
   - Have you made changes along the way?
   - Have there been any particular challenges?
   - Has anything gone more smoothly than expected?
   - If you had to go back and do it all again, is there anything you would you do differently?
   - Do you think your team had the capacity to readily take this on?

**Experiences of returning additional findings**

1. Can you tell me about your experience when giving results to patients?
   - What type of results have you returned?
   - Were there any cases that stand out for you as particularly challenging or particularly beneficial for the patient/family?
   - Do most patients recall opting for additional findings?
   - Do patients generally understand what their results mean for their health? (e.g. whether this means they will develop the condition later on or not)
   - How long does the discussion about their additional finding results last?
2. How do patients react/respond to their additional findings result?
   - What type of questions do patients ask?
   - What concerns do they have?
   - Has returning additional findings results to patients raised any concerns for you? (induced anxiety in patient, patient’s mental health, timing of result)
3. What information do you provide to your patients regarding next steps?
   - Further information about referrals to specialists
   - Information about support groups
   - Referral to psychosocial support e.g. genetic counselling/clinical psychology/counsellors
   - Lifestyle advice (diet, exercise, cessation of smoking, etc)
   - Written information/pamphlets
4. What advice and support do you give to patients regarding communicating their additional findings result with family members?

**Offering additional findings routinely in the NHS Genomic Medicine Service**

1. How do you feel about offering looked for additional findings in routine clinical practice as part of the NHS Genomic Medicine Service?
   - Who should be offered additional findings? e.g All adults offered genomics sequencing tests?

- How would you feel about it being offered in acute care settings?
  - What additional findings should be reported?
  - Do you think the ACMG list is an appropriate guide to follow in the UK, why/not?

1. What do you think the main challenges would be if additional findings were routinely offered to people undergoing genomic sequencing?
2. What lessons from offering additional findings in the 100,000 Genomes Project would you bring to offering additional findings in routine clinical practice?
3. What can we do to support patients/parents with their decision making if they were offered looked for additional findings?
4. What approach to consent would you use if looked for additional findings were offered routinely?
5. Who do you think should discuss additional findings results with patients? For example, should it be the genetics team, the healthcare professional who made the referral, or the healthcare professional within the relevant speciality e.g. oncology?
6. Do you have any final thoughts about additional findings?
